# Frequency of detection and load of amastigotes in the pancreas of *Leishmania infantum*-seropositive dogs: clinical signs and histological changes

**DOI:** 10.1186/s13071-021-04813-3

**Published:** 2021-06-12

**Authors:** William de Oliveira Kost, Sandro Antonio Pereira, Fabiano Borges Figueiredo, Artur Augusto Velho Mendes Junior, Maria de Fátima Madeira, Luciana de Freitas Campos Miranda, Raquel de Vasconcellos Carvalhaes de Oliveira, Luiz Cláudio Ferreira, Fernanda Nazaré Morgado, Rodrigo Caldas Menezes

**Affiliations:** 1 Laboratório de Pesquisa Clínica em Dermatozoonoses em Animais Domésticos, Instituto Nacional de Infectologia Evandro Chagas, Fundação Oswaldo Cruz, Av. Brasil, 4365, Rio de Janeiro, RJ 21040-360 Brazil; 2 Instituto Carlos Chagas, Fundação Oswaldo Cruz, Rua Professor Algacyr Munhoz Mader, 3775, Curitiba, PR 81350-010 Brazil; 3 Laboratório de Pesquisa Clínica e Vigilância em Leishmanioses, Instituto Nacional de Infectologia Evandro Chagas, Fundação Oswaldo Cruz, Av. Brasil, 4365, Rio de Janeiro, RJ 21040-360 Brazil; 4 Laboratório de Epidemiologia Clínica, Instituto Nacional de Infectologia Evandro Chagas, Fundação Oswaldo Cruz, Av. Brasil, 4036, Rio de Janeiro, RJ 21040-361 Brazil; 5 Serviço de Anatomia Patológica, Instituto Nacional de Infectologia Evandro Chagas, Fundação Oswaldo Cruz, Av. Brasil, 4365, Rio de Janeiro, RJ 21040-360 Brazil; 6 Laboratório de Imunoparasitologia, Instituto Oswaldo Cruz, Fundação Oswaldo Cruz, Av. Brasil, 4365, Rio de Janeiro, RJ 21040-360 Brazil

**Keywords:** Canine visceral leishmaniasis, Histopathology, Pancreatitis, Immunohistochemistry

## Abstract

**Background:**

Zoonotic visceral leishmaniasis is caused by the protozoan *Leishmania infantum* and is highly lethal in humans and dogs if left untreated. The frequency of this parasite and associated histological changes in the pancreas of dogs are poorly studied. Therefore, the objectives of this study were to evaluate the frequency of detection and load of amastigotes in the pancreas of *L. infantum*-seropositive dogs and to identify the clinical signs and histological changes associated with parasitism of this organ.

**Methods:**

One hundred forty-three dogs from an endemic area in Brazil that tested seropositive for *L. infantum* were studied. The dogs were clinically examined, killed, and necropsied between 2013 and 2014. One fragment of the pancreas was randomly collected for histopathology and immunohistochemistry, and spleen and bone marrow were collected for culture.

**Results:**

*Leishmania* amastigotes were detected in the pancreas of 22 dogs (15.4%) by immunohistochemistry, all exhibiting *L. infantum* parasitism in the spleen and/or bone marrow. Poor body condition and cachexia were only associated with infection of the pancreas with *Leishmania* spp. (*p* = 0.021) and were found in 40.9% of dogs with pancreatic infection. Anorexia, vomiting, and/or diarrhea were observed in 9.2% of dogs with pancreatitis. The median parasite load in the pancreas was 1.4 infected macrophages/mm^2^. Pancreatic histological changes and their frequencies were: granulomatous pancreatitis (28.0%), lymphoplasmacytic pancreatitis (23.8%), acinar cell degeneration (6.3%), fibrosis (5.6%), hemorrhage (2.1%), eosinophilic pancreatitis (0.7%), suppurative pancreatitis (0.7%), and necrosis (0.7%).

**Conclusions:**

The present results demonstrate that *L. infantum* is one of the etiological agents of chronic pancreatitis in dogs; however, the frequency of detection and parasite load are low in this organ. The lack of an association of poor body condition and cachexia with pancreatitis and the low frequency of clinical signs commonly associated with pancreatitis suggest that a significant portion of the organ is not affected by this parasite. On the other hand, the association of poor body condition and cachexia with concomitant infection of the pancreas, spleen, and/or bone marrow with this parasite suggests that these manifestations are the result of a more advanced stage of canine visceral leishmaniasis.

**Graphic abstract:**

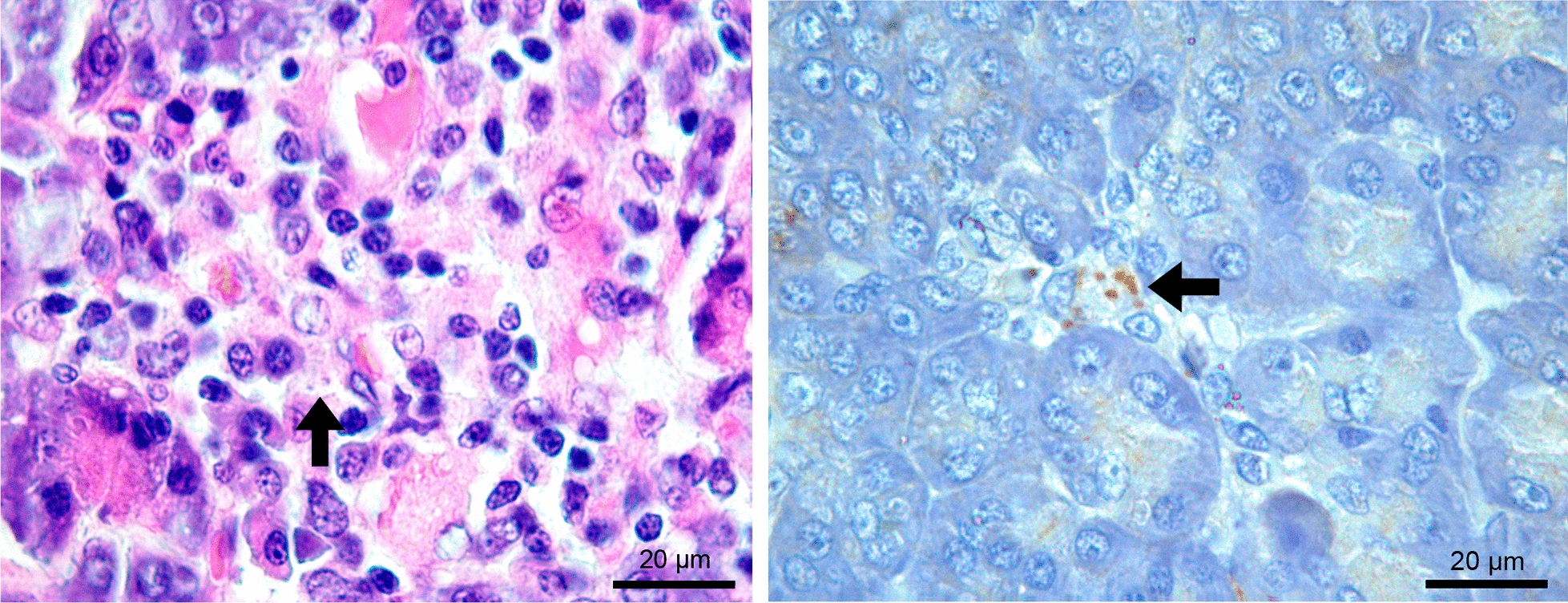

## Background

Zoonotic visceral leishmaniasis (ZVL) affects humans and domestic and wild mammals. This disease is caused by the protozoan *Leishmania infantum* (syn. *L. chagasi*), and the domestic dog (*Canis familiaris*) is the main reservoir of this parasite in urban areas [1]. In Brazil, the vectors responsible for the transmission of this protozoan are female sand flies of the species *Lutzomyia longipalpis* and *Lutzomyia cruzi* [2]. Zoonotic visceral leishmaniasis is spreading in Brazil and in the state of Rio de Janeiro [3, 4]. There were about 20,000 human cases of ZVL in Brazil between 2014 and 2019 [5]. In dogs infected with *L. infantum*, the multiplication of this parasite in macrophages causes the destruction of these cells and can induce an immune response of the Th1/Th2 type. This immune response leads to tissue damage in several organs mainly as a result of a granulomatous inflammatory reaction, the deposition of immune complexes, or the production of autoantibodies [6].

Inflammation of the pancreas or pancreatitis is a common alteration in dogs, which is classified as acute or chronic [7]. Acute and chronic pancreatitis is distinguished based not only on duration but also on histological changes. Unlike acute pancreatitis, which is characterized mainly by a predominance of neutrophils in the inflammatory infiltrate, edema, and necrosis, chronic pancreatitis is characterized by a predominantly mononuclear inflammatory infiltrate and irreversible changes such as atrophy and fibrosis [7, 8]. Depending on the degree of organ involvement, pancreatitis in dogs can vary from subclinical to clinical. The main clinical signs are anorexia, depression, abdominal pain, diarrhea, and vomiting, which can lead to death [8–10]. Chronic pancreatitis causes the progressive loss of exocrine and endocrine function, which can result in exocrine pancreatic insufficiency (EPI) and diabetes mellitus when > 80 to 90% of the exocrine or endocrine tissue is lost [7]. The main clinical signs of EPI are polyphagia, increased fecal volume, and weight loss that can progress to cachexia [7, 11]. Important etiologies of pancreatitis in dogs include dietary factors, hyperlipoproteinemia, drugs, toxins, hypercalcemia, pancreatic trauma, ischemia, and parasitosis, as well as idiopathic causes [7, 9, 12–14].

Reports of pancreatitis associated with *L. infantum* infection are rare. Carrasco et al. [12] reported the case of a dog with visceral leishmaniasis showing signs of acute pancreatitis. The final diagnosis after histopathological examination of the pancreas was acute hemorrhagic pancreatitis associated with ZVL, although no amastigote forms were detected in this organ. Guerra et al. [15] found *Leishmania* amastigote forms in the pancreas of 22.6% of naturally infected dogs examined by immunohistochemistry (IHC). However, these authors did not describe pancreatic histological changes. Pancreatitis is an adverse effect of drugs used to treat ZVL in dogs and humans such as meglumine antimoniate, but the mechanisms underlying pancreatitis induced by these drugs in dogs and humans are unknown [13, 16, 17]. A marked increase of serum pancreatic lipase immunoreactivity (PLI) concentration has been reported in dogs with pancreatitis caused by the treatment of ZVL with meglumine antimoniate [13, 16].

Knowledge of pancreatic parasitism with *L. infantum* in dogs and the associated clinical signs and histological changes is important for a better understanding of the pathogenesis of this parasitosis as well as to provide information for future studies on adverse effects associated with anti-*Leishmania* drugs used for the treatment of ZVL. Therefore, the aims of this study were to assess the frequency of detection and load of amastigotes in the pancreas of *L. infantum*-seropositive dogs and to identify the clinical signs and histological changes associated with the parasite in this organ.

## Methods

### Sample

This is a retrospective study of a non-probabilistic sample of 143 pancreata obtained from dogs that tested seropositive for anti-*Leishmania* antibodies by the rapid dual-path platform assay (TR DPP®) [18] and by enzyme immunoassay (ELISA) [19]. Both serological tests are produced by BioManguinhos (Fiocruz, Rio de Janeiro, Brazil). These two serological tests and diagnostic criteria were used because they are accurate [20] and are recommended by the Brazilian Ministry of Health for classification of infection of dogs with *L. infantum* and for euthanasia of the animals as a control measure of canine visceral leishmaniasis [2]. All dogs included in the study had owners, were domiciled, and did not receive any treatment for ZVL. The serological tests were performed by public health services participating in the ZVL surveillance and control program of the state of Rio de Janeiro, with permission of the owners. The samples were collected between 2012 and 2013 from dogs from the town of Barra Mansa (22°32′25.19″ S and 44°10′35.33″ W), located in the south of the state of Rio de Janeiro. This town is an endemic region for ZVL, including human and canine cases [21, 22]. Since the dogs tested positive, they were sent by the Municipal Health Department of Barra Mansa to be killed at the Evandro Chagas National Institute of Infectious Diseases (INI-Fiocruz).

At INI-Fiocruz, the dogs were examined clinically by inspecting the skin and mucous membranes (oral and ocular) and by palpation of the superficial lymph nodes and abdominal organs. The following clinical signs of ZVL in dogs were considered: skin changes (desquamation, dull hair coat, onychogryphosis, alopecia, and cutaneous ulcer), poor body condition, cachexia, lymphadenomegaly, splenomegaly, hepatomegaly, pale ocular or oral mucosae, and keratoconjunctivitis [23]. A poor body condition was classified when the dog had easily visible ribs, lumbar vertebrae, and pelvic bones, some evidence of other bony prominences, no palpable body fat, and absent or minimal to obvious loss of muscle mass. Cachexia was defined when the animal’s ribs, lumbar vertebrae, pelvic bones, and all bony prominences were evident from a distance, with no discernible body fat and severe loss of muscle mass. The dogs were then euthanized with an intravenous overdose of sodium thiopental and potassium chloride in accordance with the recommendations of the national ZVL control program [2] and the guidelines of the Federal Council of Veterinary Medicine [24]. The frequencies of anorexia, vomiting, and/or diarrhea, which are commonly associated with pancreatitis in dogs, were obtained from the medical records of the Municipal Health Department of Barra Mansa.

During necropsy, the pancreas was examined macroscopically, and one fragment of this organ with a maximum length of 1.0 to 2.0 cm and 0.3 to 0.5 cm thick was randomly collected, fixed in 10% buffered formalin, and processed for paraffin embedding [25] for histopathology and IHC (detection of amastigote forms of *Leishmania* spp.). Additionally, fragments of spleen and bone marrow were collected aseptically and immersed in sterile saline solution containing antimicrobials for parasitological culture.

### Histopathology and immunohistochemistry

Three serial 5-µm histological sections were cut from paraffin blocks containing pancreatic tissue samples stored at the Pathological Anatomy Service of the Evandro Chagas National Institute of Infectious Diseases, Oswaldo Cruz Foundation. Two sections were mounted on non-silanized slides for histopathology, and one section was mounted on silanized slides for IHC.

For histopathology, the tissues were stained with hematoxylin–eosin [25], and the inflammatory infiltrate was classified as follows: granulomatous, predominance of cells of the monocyte-macrophage system (activated macrophages, epithelioid macrophages, or multinucleate giant cells); lymphoplasmacytic, predominance of lymphocytes, and plasma cells; suppurative, predominance of neutrophils; eosinophilic, predominance of eosinophils. Pancreatitis was classified as acute to subacute if the inflammatory infiltrate exhibited a predominance of neutrophils or eosinophils, with or without edema and necrosis, and no irreversible changes (atrophy or fibrosis); chronic, if characterized by a predominantly mononuclear infiltrate (granulomatous or lymphoplasmacytic) with or without irreversible changes such as atrophy and fibrosis [7, 8].

For evaluation of the intensity of inflammation, cell types (macrophages, plasma cells, lymphocytes, eosinophils, and neutrophils) detected in the inflammatory infiltrate were analyzed semi-quantitatively under a light microscope using a 1-mm^2^ optical grid and a manual cell counter. The number of cells was determined in one microscopic field at 400 × magnification in the most cellular area of the histological section. The median number of cells was calculated for the entire inflammatory infiltrate (sum of all cell types found). The intensity of the inflammatory infiltrate was classified as absent, mild (1 to 250 inflammatory cells/mm^2^), moderate (> 250 and ≤ 450 inflammatory cells/mm^2^), and severe (> 450 inflammatory cells/mm^2^).

For IHC aimed at detecting amastigote forms of *Leishmania* spp., the tissues were submitted to the steps of deparaffinization, rehydration, blocking of endogenous peroxidase, antigen retrieval, blockade of nonspecific protein binding, and incubation with polyclonal rabbit anti-*Leishmania* serum diluted 1:500, following a previously described protocol [26]. The polymer-based HiDef Detection HRP™ Polymer System (Cell Marque, Rocklin, CA, USA) was used for the detection of *Leishmania* amastigote forms according to manufacturer's recommendations. Histological sections of organs intensely parasitized with *Leishmania* amastigotes were incubated with non-immune homologous serum as negative control and with polyclonal rabbit anti-*Leishmania* serum as positive control.

For the evaluation of parasite load in the pancreas by IHC, macrophages parasitized with *Leishmania* amastigote forms were quantified as described for the quantification of inflammatory cells by histopathology. However, cells were counted in five fields at 400 × magnification in the most parasitized areas of the sections. The average number of parasitized macrophages was calculated, and the parasite load was classified as absent, low (0.2 to 10 parasitized macrophages/mm^2^), and moderate to intense (> 10 parasitized macrophages/mm^2^).

### Parasitological culture and identification of *Leishmania* species

The fragments were cultured at 26–28°C in Novy-MacNeal-Nicolle medium plus Schneider’s Drosophila medium (Sigma-Aldrich®, St. Louis, MO, USA) supplemented with 10% fetal bovine serum and penicillin and streptomycin as antibiotics [27]. Parasites isolated in culture were identified as *L. infantum* by multilocus enzyme electrophoresis [28].

### Statistical analysis

For exploratory analysis, the relative frequencies of the categorical variables were calculated. For quantitative variables, medians and their interquartile range were used. The normality of the quantitative variables was rejected by the Shapiro-Wilk test. Pearson’s chi-square test and Fisher’s exact test in the case of expected counts < 5 or 2 × 2 tables were used to compare the occurrence of clinical signs of poor body condition and cachexia and the different histological changes as well as to associate the frequencies of histological changes with the results of IHC for the diagnosis of *Leishmania* spp. The Mann-Whitney test was used to compare the intensity of the inflammatory infiltrate (for all cells) between *Leishmania* spp.-positive and -negative pancreas. Spearman’s correlation test was applied to verify the correlation between parasite load and the intensity of the inflammatory infiltrate in the pancreas, where values close to + 1 indicate a positive correlation, close to − 1 a negative correlation, and 0 no correlation.

The tests were considered significant at the 5% level. Despite criticism of the exclusive use of *p*-values and due to the small sample size in some groups, the theoretical relevance and a difference of at least 10 percentage points were also considered to be indicative of a potential difference between groups [29]. In addition, 95% confidence intervals (95% CI) were provided for proportions as a measure of uncertainty. The analysis was performed using the R software, version 3.5.1 [30].

## Results

Of the 143 dogs evaluated, 75 (52.4%) were male and 118 (82.5%) were mongrel. Five of the 25 breed dogs were Labrador Retrievers, four were Pinschers, three were American Pit Bull Terriers, three were Dachshunds, two were Cocker Spaniels, two were Rottweilers, two were German Shepherds, one was a Dobermann, one was a Canadian Shepherd, one was a Poodle, and one was a Cane Corso. The age of the dogs ranged from 1 to 7 years in 102 (71.3%) animals, 32 (22.4%) were older than 7 years, and nine (6.3%) were up to 12 months old. Eighty-two animals (57.3%) had at least one clinical sign compatible with ZVL, and 61 (42.7%) had no clinical signs compatible with ZVL. Anorexia, vomiting, and/or diarrhea were observed in nine (6.3%) dogs, three of them without clinical signs compatible with ZVL. The clinical signs observed are described in Table 1.

**Table 1:** Frequency of clinical signs in 143 dogs from the town of Barra Mansa, Rio de Janeiro, Brazil, that tested seropositive for anti-*Leishmania* spp. antibodies (2012 to 2013)

Clinical signs (*n* = 143)	*n* (%)
Skin changes	40 (28.0)
Hepato-/splenomegaly	40 (28.0)
Poor body condition/cachexia	30 (21.0)
Lymphadenomegaly	23 (16.1)
Keratoconjunctivitis	18 (12.6)
Diarrhea	9 (6.3)
Anorexia	5 (3.5)
Vomiting	3 (2.1)

*n*, number of dogs

**Fig. 1:**
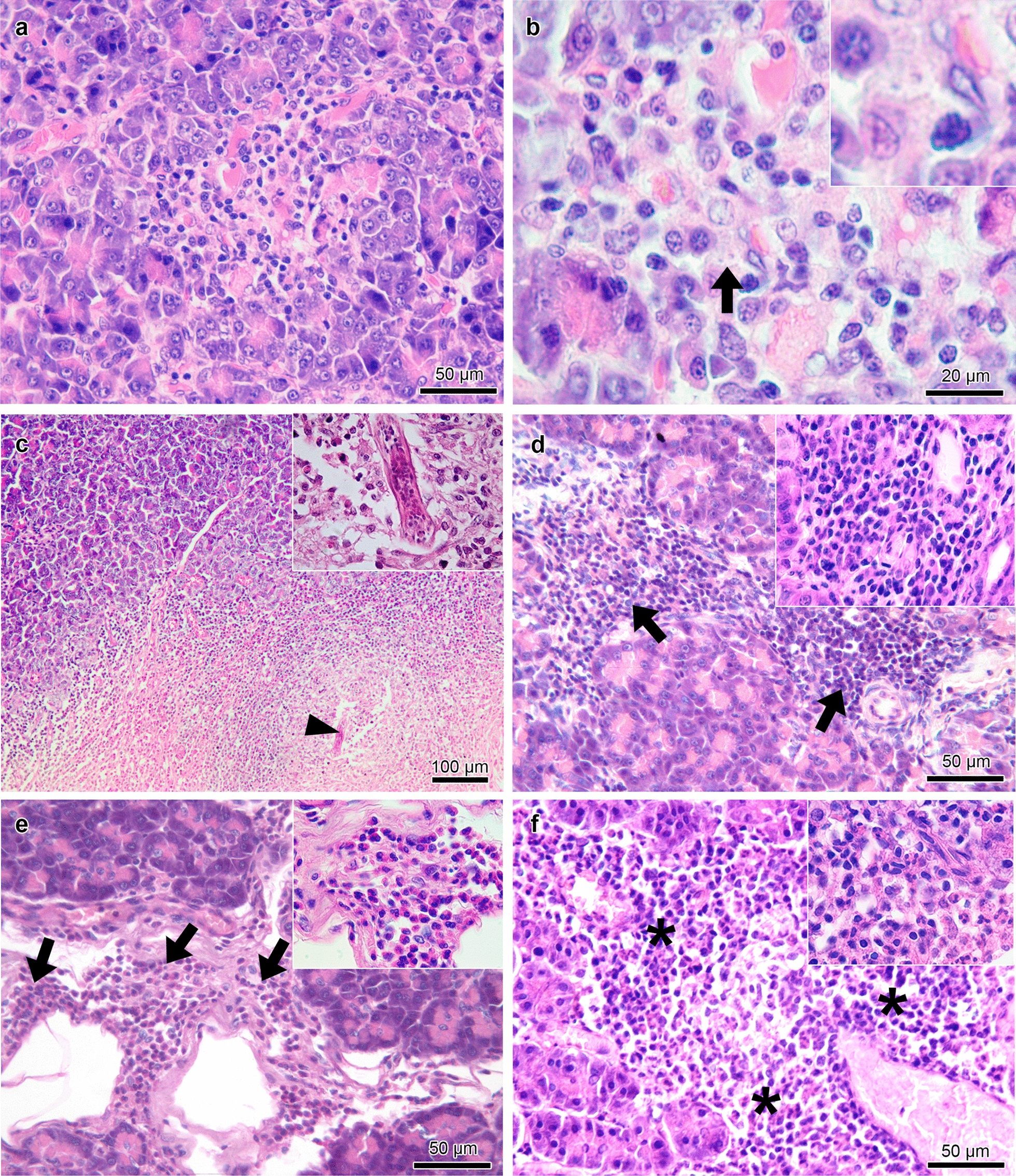
Histological changes in the pancreas of dogs seropositive for anti-*Leishmania infantum* antibodies with chronic (**a**–**d**) and acute to subacute (**e**–**f**) pancreatitis. **a** Moderate diffuse granulomatous pancreatitis composed mainly of macrophages, with a smaller number of plasma cells and lymphocytes. **b** Higher magnification of the previous figure showing many macrophages, with a smaller number of plasma cells and lymphocytes and *Leishmania* spp. amastigotes (arrow and inset) within the cytoplasm of a macrophage in the interstitial tissue amid the inflammatory infiltrate. **c** Severe granulomatous pancreatitis composed mainly of macrophages, with a smaller number of lymphocytes, plasma cells, and eosinophils. Fibrosis and a nematode larva (arrowhead and inset) are also seen in the parenchyma. **d** Severe interstitial lymphoplasmacytic pancreatitis (arrows and inset) consisting mainly of lymphocytes and plasma cells. **e** Severe perivascular eosinophilic pancreatitis (arrows and inset) composed mainly of eosinophils, with few lymphocytes and plasma cells. **f** Severe perivascular suppurative pancreatitis (asterisks and inset) consisting mainly of neutrophils, with many lymphocytes and plasma cells and few macrophages. Hematoxylin–eosin staining **(a**–**f)**

**Fig. 2:**
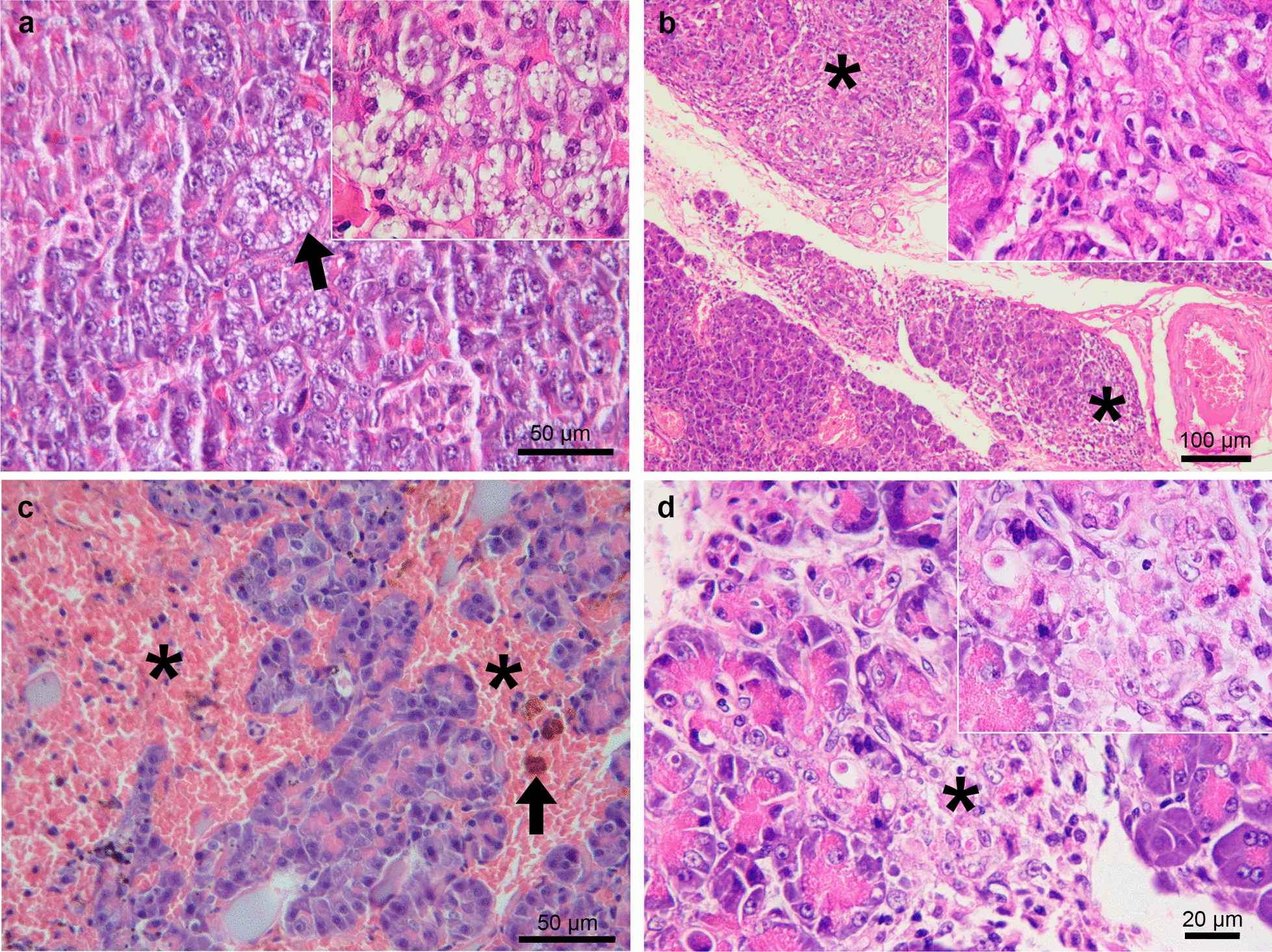
Histological changes in the pancreas of dogs seropositive for anti-*L. infantum* antibodies. **a** Vacuolar and diffuse acinar cell degeneration (arrow and inset). **b** Diffuse fibrosis in the parenchyma (asterisks and inset). **c** Extensive hemorrhagic area (asterisks) with hemosiderin (arrow) in the parenchyma. **d** Discrete focus of necrosis in the parenchyma (asterisk and inset). Hematoxylin–eosin staining (**a**–**d**)

No gross changes were observed in the examined pancreas. However, this organ exhibited histological changes in 80 (55.9%) of the 143 dogs examined, and pancreatitis was observed in 76 (53.1%). Seventy-four (97%) of the 76 cases of pancreatitis were chronic (granulomatous or lymphoplasmacytic) (Fig. 1a–d), and 2 (3%) were acute to subacute (suppurative or eosinophilic) (Fig. 1e, f). Among the 80 dogs with histological changes in the pancreas, 4 (5%) did not have pancreatitis. Two of these four dogs had only vacuolar degeneration of acinar cells (Fig. 2a), and two had only fibrosis. The frequencies of the types of pancreatitis and other histological changes are shown in Table 2.

**Table 2:** Frequency of pancreatic histological changes observed in 143 dogs from the town of Barra Mansa, Rio de Janeiro, Brazil, that tested seropositive for anti-*Leishmania* antibodies (2012 to 2013)

Pancreatic histological changes (*N* = 143)	*n*	%
Granulomatous pancreatitis	40	28.0
Lymphoplasmacytic pancreatitis	34	23.8
Vacuolar degeneration of acinar cells	7	4.9
Fibrosis	6	4.2
Hemorrhage	3	2.1
Eosinophilic pancreatitis	1	0.7
Suppurative pancreatitis	1	0.7
Necrosis	1	0.7
No alterations	63	44.1

*N*, total number of examined dogs; *n*, number of dogs with each type of pancreatic histological alteration

**Fig. 3:**
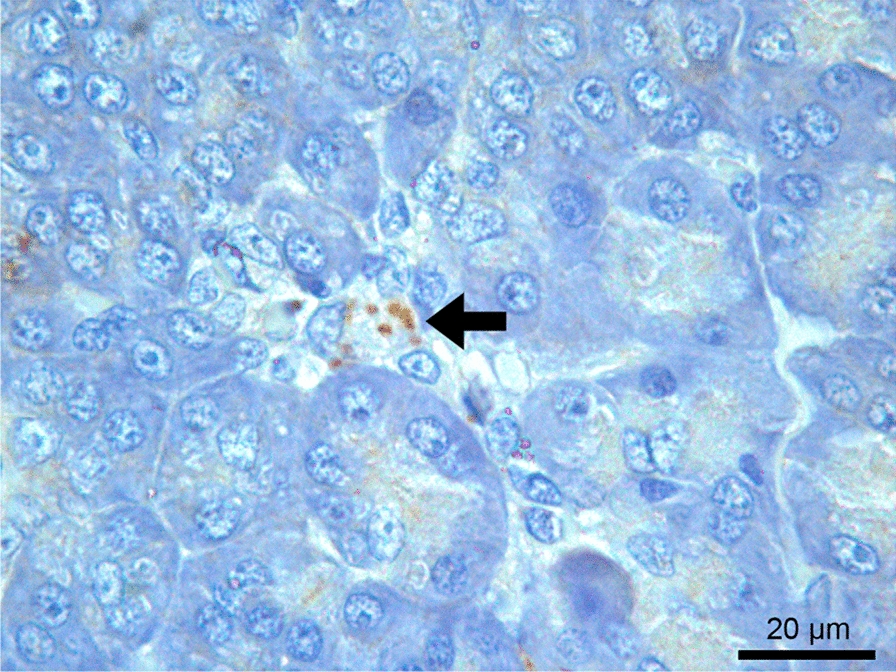
Pancreas of a dog seropositive for anti-*Leishmania infantum* antibodies with mild granulomatous inflammation. Brown-stained *Leishmania* spp. amastigotes in the cytoplasm of macrophages (arrow) in the interstitial tissue. Immunohistochemistry

Amastigote forms of *Leishmania* spp. were detected by IHC in the pancreas of 22 dogs (15.4%) (Fig. 3). All six cases that tested positive for *Leishmania* spp. amastigote forms by histopathology were also positive by IHC, which detected an additional 16 cases.

Table 3 shows the dog breed, sex, and age according to the result of IHC for the diagnosis of *Leishmania* spp. in the pancreas. The four dog breeds that tested positive by IHC included two Labrador Retrievers, one Pinscher, and one Cocker Spaniel.

**Table 3:** Characteristics of dogs according to the result of IHC for the detection of *Leishmania* spp. amastigote forms in the pancreas of 143 dogs from the town of Barra Mansa, Rio de Janeiro (2012 to 2013)

Characteristics of dogs	IHC for detection of *Leishmania* amastigotes						*p* value
	Negative (*N* = 121)			Positive (*N* = 22)			
	*n*	%	95% CI (%)	*n*	%	95% CI (%)	
Mongrel	100	82.6	74.7–88.9	18	81.8	59.7–94.8	1.000
Breed	21	17.4	11.1–25.3	4	18.2	5.2–40.3	
Male	58	47.9	38.8–57.2	17	77.3	54.6–92.2	0.019^a^
Female	63	52.1	42.8–61.2	5	22.7	7.8–45.4	
Up to 12 months old	8	6.6	2.9–12.6	1	4.5	0.1–22.8	0.846
1 to 7 years old	87	71.9	63.0–79.7	15	68.2	45.1–86.1	
Older than 7 years	26	21.5	14.5–29.9	6	27.3	10.7–50.2	

*N* number of dogs, *n* number of samples, *IHC* immunohistochemistry, *95% CI* 95% confidence interval

^a^Statistically significant difference at the 5% level

Among the 76 dogs with pancreatitis, 61 were mongrels (32 with granulomatous pancreatitis, 28 with lymphoplasmacytic pancreatitis, and 1 with eosinophilic pancreatitis), 4 were Labrador Retrievers (3 with granulomatous pancreatitis and 1 with lymphoplasmacytic pancreatitis), 3 were Pinschers (3 with granulomatous pancreatitis), 2 were American Pitbull Terriers (2 with granulomatous pancreatitis), 2 were Cocker Spaniels (1 with granulomatous pancreatitis and 1 with lymphoplasmacytic pancreatitis), 1 was a Rottweiler (with granulomatous pancreatitis), 1 was a German Shepherd (with granulomatous pancreatitis), 1 was a Canadian Shepherd (with granulomatous pancreatitis), and 1 was a Dachshund (with granulomatous pancreatitis). Thirty-six were males, and 40 were females. The frequencies of pancreatitis according to age were: 5 of 9 (55.6%) dogs up to 12 months old, 50 of 102 (49.0%) dogs between 1 and 7 years old, and 21 of 32 (65.6%) dogs older than 7 years. There was no statistically significant association of breed, age, or sex with the presence or absence of histological changes in the examined pancreas.

Histological changes were observed in 20 (90.9%) of the 22 pancreata that tested positive for amastigote forms of *Leishmania* spp. by IHC. Chronic pancreatitis was observed in 20 (90.9%) cases, and acute to subacute pancreatitis was absent (Table 4). Among the 121 pancreata that tested negative for *Leishmania* spp. amastigote forms by IHC, pancreatic histological changes were detected in 60 (49.6%). Chronic pancreatitis was observed in 54 (44.6%) cases, acute to subacute pancreatitis in 2 (1.6%), only degeneration of acinar cells in 2 (1.6%), and only fibrosis in 2 (1.6%) (Table 4).

**Table 4:** Frequency of pancreatic histological changes according to the result of IHC for the detection of *Leishmania* spp. amastigote forms in 143 dogs from the town of Barra Mansa, Rio de Janeiro, Brazil (2012 to 2013)

Histological changes	IHC for detection of *Leishmania* amastigotes						*p* value
	Negative (*N* = 121)			Positive (*N* = 22)			
	*n*	%	95% CI (%)	*n*	%	95% CI (%)	
Granulomatous pancreatitis	23	19.0	12.4–27.1	17	77.3	54.6–92.2	0.007^a^
Lymphoplasmacytic pancreatitis	31	25.6	18.1–34.3	3	13.6	2.9–34.9	0.004^a^
Vacuolar degeneration of acinar cells	6	5.0	1.8–10.5	1	4.5	0.1–22.8	1.000
Fibrosis	4	3.3	0.9–8.2	2	9.1	1.1–29.5	0.248
Hemorrhage	2	1.6	0.2–7.4	1	4.5	0.1–22.8	1.000
Eosinophilic pancreatitis	1	0.8	0.0–4.5	0	0	–	_^b^
Suppurative pancreatitis	1	0.8	0.0–4.5	0	0	–	_^b^
Necrosis	1	0.8	0.0–4.5	0	0	–	_^b^
Absence of histological changes	61	50.4	41.2–59.6	2	9.1	1.1–29.2	0.009^a^

*N* number of dogs, *n* number of samples, *IHC* immunohistochemistry, *95% CI* 95% confidence interval

^a^Statistically significant difference at the 5% level

^b^Statistical analysis was not possible because of the absence of the histological change in one of the groups

Considering the cases of chronic pancreatitis, an interstitial inflammatory infiltrate mainly consisting of activated macrophages and a smaller number of plasma cells and lymphocytes (Fig. 1a, b) was observed in dogs with granulomatous pancreatitis (*n* = 40). In six of these cases (15%), amastigote forms of *Leishmania* spp. were detected inside the parasitophorous vacuoles in the cytoplasm of macrophages located in the interstitial tissue (Fig. 1b) and in connective tissue around the interlobular ducts. A nematode larva was observed in the parenchyma of one male dog, a 12-month-old Canadian Shepherd (Fig. 1c). This case, which was negative for *Leishmania* spp. amastigotes in the pancreas, exhibited an inflammatory infiltrate with a predominance of macrophages accompanied by many eosinophils, lymphocytes, and plasma cells as well as fibrosis (Fig. 1c). The intensity of the inflammatory infiltrate in the cases of granulomatous pancreatitis was mild in 32 (80%), moderate in 6 (15%), and severe in 2 (5%). Fibrosis (Fig. 2b) associated with an inflammatory infiltrate of moderate to severe intensity was observed in four cases. Additionally, there were two cases of hemorrhage associated with a mild inflammatory infiltrate and one case of degeneration of acinar cells associated with a moderate inflammatory infiltrate.

A predominantly perivascular multifocal inflammatory infiltrate was observed in the cases of lymphoplasmacytic pancreatitis (*n* = 34), which consisted mainly of lymphocytes and plasma cells and a smaller number of macrophages (Fig. 1d). The intensity of the inflammatory infiltrate in cases of lymphoplasmacytic pancreatitis was mild in 21 (62%), moderate in 11 (32%), and severe in 2 (6%). Additionally, one case of hemorrhage (Fig. 2c) and one case of necrosis (Fig. 2d) associated with a moderate inflammatory infiltrate were observed as well as three cases of degeneration of acinar cells associated with a moderate to severe inflammatory infiltrate.

Among the cases of acute to subacute pancreatitis, there was a predominantly perivascular multifocal inflammatory infiltrate of moderate intensity in eosinophilic pancreatitis (*n* = 1), which consisted mainly of eosinophils and a smaller number of lymphocytes and plasma cells (Fig. 1e). In suppurative pancreatitis (*n* = 1), a predominantly perivascular multifocal inflammatory infiltrate of moderate intensity was observed, mostly consisting of neutrophils with many lymphocytes and plasma cells and few macrophages (Fig. 1f).

Among the 22 dogs whose pancreas was positive for *Leishmania* spp. amastigotes by IHC, 13 (59.1%) had at least one clinical sign compatible with ZVL and 2 (9.1%) had anorexia, vomiting, and/or diarrhea. On the other hand, among the 121 dogs whose pancreas tested negative for *Leishmania* spp. amastigotes by IHC, 69 (57.0%) showed clinical signs compatible with ZVL and 7 (5.8%) had anorexia, vomiting, and/or diarrhea. The clinical signs and their frequencies in these two groups of dogs are shown in Table 5.

**Table 5:** Frequency of clinical signs according to the result of IHC for the detection of *Leishmania* spp. amastigote forms in 143 dogs from the town of Barra Mansa, Rio de Janeiro, Brazil (2012 to 2013)

Clinical signs	IHC for detection of *Leishmania* amastigotes						*p* value
	Negative (*N* = 121)			Positive (*N* = 22)			
	*n*	%	95% CI (%)	*n*	%	95% CI (%)	
Poor body condition/cachexia	21	17.4	11.1–0.2	9	40.9	20.7–63.6	0.021^a^
Skin changes	33	27.3	19.6–36.1	7	31.8	13.9–54.9	0.797
Hepato-/splenomegaly	34	28.1	20.3–37.0	6	27.3	10.7–50.2	1.000
Lymphadenomegaly	18	14.9	9.1–22.5	5	22.7	7.8–45.4	0.353
Keratoconjunctivitis	14	11.6	6.5–18.6	4	18.2	5.2–40.3	0.482
Diarrhea	7	5.8	2.3–11.6	2	9.1	1.1–29.2	0.629
Anorexia	4	3.3	0.9–8.2	1	4.5	0.1–22.8	0.572
Vomiting	3	2.5	0.5–7.1	0	0.0	0.0–15.5	_^b^
Absence of clinical signs	50	41.3	32.4–50.6	8	36.4	17.2–59.3	0.814

*N* number of dogs, *n* number of samples, *IHC* immunohistochemistry, *95% CI* 95% confidence interval

^a^Statistically significant difference at the 5% level

^b^Statistical analysis was not possible because of the absence of vomiting in one of the groups

The frequency of pancreatitis (granulomatous, lymphoplasmacytic, eosinophilic, or suppurative) was 53.3% in dogs with poor body condition or cachexia and 53.1% in dogs without these clinical signs. There was no statistically significant association between pancreatitis and poor body condition or cachexia (*p* = 1.000).

Seven (9.2%) of the 76 dogs with pancreatitis had anorexia, vomiting, and/or diarrhea. In these seven dogs, two had mild granulomatous pancreatitis and five had mild (*n* = 3) or severe (*n* = 2) lymphoplasmacytic pancreatitis. Among the 67 dogs without pancreatitis, 2 (3.0%) had anorexia, vomiting, and/or diarrhea. Pancreatitis was not significantly associated with anorexia, vomiting, and/or diarrhea (*p* = 0.174).

The median number of inflammatory cells/mm^2^ was 175.0 (46 to 807) in dogs with pancreatitis (*n* = 76). In dogs whose pancreas was positive for *Leishmania* spp. amastigotes by IHC and that simultaneously had pancreatitis (*n* = 20), the median number of inflammatory cells/mm^2^ was 167.5 (89 to 375). This number was 172 inflammatory cells/mm^2^ (46 to 807) in dogs with pancreatitis and negative for amastigote forms of *Leishmania* spp. by IHC (*n* = 56). There was no statistically significant difference in the intensity of the inflammatory infiltrate between dogs with pancreatitis that tested positive and negative for *Leishmania* spp. amastigotes by IHC (*p* = 0.881).

The load of *Leishmania* spp. amastigotes detected by IHC in the pancreas was classified as low, with a median number of 1.4 (0.4 to 7.2) infected macrophages/mm^2^. The median parasite load was 2.0 (0.4 to 7.2) and 0.6 (0.4 to 7.0) infected macrophages/mm^2^ in dogs with and without clinical signs, respectively (*p* = 0.412). In dogs positive for *Leishmania* spp. by IHC, the median parasite load was 3.8 (0.6 to 7.2) infected macrophages/mm^2^ in those with clinical signs of poor body condition or cachexia (*n* = 9) and 0.6 (0.6 to 7.0) infected macrophages/mm^2^ in those without these clinical signs (*n* = 13) (*p* = 0.272).

Of the 143 dogs included in this study, 115 (80.4%) were positive for *L. infantum* in spleen and/or bone marrow by culture. The spleen and bone marrow were positive in 79 (68.7%) dogs, only the spleen in 22 (19.1%), and only bone marrow in 14 (12.2%). All 22 animals whose pancreas was positive for *Leishmania* sp. amastigote forms by IHC tested positive for *L. infantum* in the spleen and/or bone marrow by culture. Four (18.1%) of these 22 dogs tested positive by culture in the spleen and 18 (81.9%) in the spleen and bone marrow. Of the 121 dogs whose pancreas was negative for *Leishmania* spp. amastigote forms by IHC, 94 (77.7%) were positive for *L. infantum* in the spleen and/or bone marrow by culture. Analysis of the frequency of *L. infantum* culture positivity in spleen and/or bone marrow showed a difference of 30.9 percentage points between the group of 30 dogs with poor body condition or cachexia and the group of 113 dogs without these clinical signs (96.6% and 65.7%, respectively), although the test indicated no statistical significance (*p* = 0.441).

## Discussion

The frequency of *Leishmania* spp. amastigotes in the pancreas observed in the present study was slightly lower than the frequency of 22.6% reported by Guerra et al. [15] using IHC. However, this frequency was much lower than that obtained by IHC in other organs of dogs that tested seropositive for anti-*L. infantum* antibodies by TR DPP® and ELISA, such as spleen (78.79 to 81.5%), bone marrow (64.6%), liver (73.6%), lymph node (76.9 to 81.5%), skin (45.6 to 65.4%), and genital tract (85.0 to 90.0%) [15, 26, 31]. Unlike organs of the mononuclear phagocyte system, skin, and genital tract, these results suggest the absence of tropism of *L. infantum* for the pancreas [15, 26, 31]. On the other hand, the frequency of pancreatic infection might be higher than that found in this study since the sensitivity of IHC for the diagnosis of *L. infantum* infection in tissues of dogs is lower than that of the polymerase chain reaction [32, 33]. Further studies using molecular techniques are needed to confirm this hypothesis.

The frequency of *Leishmania* spp. positivity in the pancreas by IHC was 54.6 percentage points higher in males compared to females. This result differs from that of other authors who found no sex predilection of infection with *L. infantum* [34–36]. Although the difference in pancreatic infection with *Leishmania* spp. between males and females was statistically significant, the small number of dogs with pancreatic infection may have influenced the result. Therefore, future studies involving a larger sample of dogs with a positive pancreas are necessary to confirm that males have a greater predisposition to pancreatic infection with *L*. *infantum*.

The frequency of chronic pancreatitis was high in the dogs studied, which was the most common alteration. In a retrospective study (2002–2007) of pancreatic diseases in 2832 necropsied dogs from the state of Rio Grande do Sul, Brazil, pancreatitis was also the most frequent disease [37]. However, the frequency of pancreatitis (2%) was much lower than that observed in the present study and acute pancreatitis (7%) was more common than chronic pancreatitis (5%) in dogs with pancreatic alterations [37]. A study conducted in Scotland on 200 dogs [38] also identified chronic pancreatitis as the most common alteration, but its frequency (25.5%) was half the value found in the present study. The dogs analyzed by Marcato et al. [37] and Watson et al. [38] were from regions where canine visceral leishmaniasis was not endemic, and the authors also did not report the presence of the parasite. Thus, the higher frequency of chronic pancreatitis in the present study and the fact that this alteration was more common in dogs whose pancreas tested positive for *Leishmania* spp. suggest the influence of this parasite on the occurrence of this histological alteration. However, only granulomatous pancreatitis was significantly associated with the presence of amastigote forms of *L. infantum*. This result corroborates the findings of other studies in which a granulomatous infiltrate was the type of inflammatory reaction associated with *L. infantum* infection in other organs such as skin, organs of the mononuclear phagocyte system, liver, genital tract, and central nervous system [6, 26, 39].

The results of the present study also suggest that lymphoplasmacytic pancreatitis may be associated with pancreatic infection caused by *L. infantum*. However, most cases of this type of pancreatitis, as well as the two cases of acute to subacute pancreatitis, occurred in the absence of this parasite, suggesting other causes of this alteration. Carrasco et al. [12] reported acute hemorrhagic pancreatitis in a dog with ZVL from Spain, suggesting that the lesions were due to vasculitis resulting from the deposition of immune complexes associated with *L. infantum* infection. These authors detected many *Leishmania* spp. amastigote forms in spleen and lymph nodes but not in the pancreas. Thus, despite the lack of detection of *Leishmania* spp. amastigotes in the pancreas of various cases of pancreatitis in the dogs studied here, the possibility that *Leishmania* spp. indirectly caused this alteration cannot be ruled out. In these cases of non-granulomatous pancreatitis and in the absence of detection of *Leishmania* spp., causes unrelated to ZVL and already described in dogs may have occurred. These possible causes include hemolytic anemia caused by babesiosis, dietary factors, hyperlipoproteinemia, ascending infection with intestinal bacteria, drug treatments, pancreatic duct obstruction, toxins, hypercalcemia, pancreatic trauma, ischemia, and idiopathic causes [7, 9, 40, 41].

Poor body condition and cachexia were the only clinical signs that differed between *Leishmania*-seropositive dogs that tested positive and negative for the presence of *Leishmania* spp. amastigote forms in the pancreas. Cachexia is a common clinical sign in dogs with ZVL [39] but its etiology is not well understood. According to Koutinas and Koutinas [6], in dogs infected with *L. infantum*, cachexia would be a consequence of proteinuria caused by glomerulonephritis related to infection with this parasite. Additionally, Pearson et al. [42] linked cachexia in hamsters with experimental visceral leishmaniasis to decreased food intake and the secretion of catabolic cytokines by splenic macrophages. However, although associated with poor body condition/cachexia, pancreatic infection with *Leishmania* spp. was probably not a direct cause of these clinical signs in the present study because of the lack of association of pancreatitis and *Leishmania* spp. load in the pancreas with poor body condition/cachexia, the low frequency of clinical signs commonly associated with pancreatitis, the low parasite load in the pancreas, as well as the mild to moderate inflammatory infiltrate in 95% of cases of pancreatitis, absence of cases of atrophy, and the few cases of fibrosis, necrosis, and degeneration in the infected pancreas. These results suggest that a significant portion of the pancreas was not compromised; hence, no EPI was present. Impairment of > 80 to 90% of the secretory capacity of the pancreas would be necessary for the occurrence of EPI associated with clinical manifestations of poor body condition/cachexia [7, 43].

One limitation of this study is the fact that it was not possible to assess the extent and severity of pancreatitis throughout the pancreas because clinical examination and histopathology of only one fragment randomly selected from this organ were performed. Oliguria, renal azotemia, severely increased hepatic enzyme activities, hypocalcemia, hypoglycemia, severe hyperglycemia, hyperkalemia, leukocytosis, shock, and disseminated intravascular coagulation are considered indicators of severe pancreatitis [9, 44]. In addition, PLI assays are the most accurate serum marker for pancreatitis [44, 45]. Therefore, new studies investigating the consequences of pancreatic infection with *Leishmania* spp. in dogs submitted to necropsy should combine histopathological analysis of multiple fragments obtained from different regions of the pancreas (left lobe, right lobe, and body), physical examination, and evaluation of the dog’s history together with serum PLI concentration, complete blood count, serum biochemistry profile, and urinalysis [44].

One possible explanation for the association of poor body condition and cachexia with *Leishmania* spp. infection of the pancreas observed in the present study might be a more advanced stage of visceral leishmaniasis. The isolation of *L*. *infantum* by parasitological culture from the spleen and/or bone marrow of all dogs with pancreatic infection in this study suggests that the involvement of this organ is the result of an advanced stage of the disease characterized by the concomitant involvement of other viscera. Carrasco et al. [12] also observed infection of other viscera such as spleen, lymph nodes, and liver in a dog with acute pancreatitis associated with *Leishmania* spp. In the case of disseminated visceral leishmaniasis, other changes that can cause poor body condition and cachexia may have occurred, such as anorexia accompanied by malnutrition and kidney damage with loss of proteins in urine [6, 46]. As possible other indicators of a more advanced stage of visceral leishmaniasis, dogs with pancreatic infection exhibited a higher frequency of clinical signs, including dermatological and ophthalmic alterations and lymphadenomegaly. The hypothesis that chronic pancreatitis associated with *L. infantum* infection can compromise the function of this organ and progress to EPI accompanied by cachexia due to the progression of ZVL cannot be completely ruled out. This hypothesis is based on the observation that chronic pancreatitis caused by parasites is associated with cachexia in animals [47] and is generally progressive in dogs [7].

Considering that pancreatitis in dogs can be caused by infection with *L. infantum*, as demonstrated in the present study, and by some drugs used for the treatment of leishmaniasis such as meglumine antimoniate [13, 16], the pancreas should be evaluated before and during treatment of canine visceral leishmaniasis. Physical examination, evaluation of the dog’s history, a complete blood count, serum biochemistry profile, and urinalysis, combined with the use of highly sensitive and specific tests such as serum PLI concentration and abdominal ultrasonography, are recommended for the diagnosis of pancreatitis in dogs [44]. If pancreatitis is detected, the use of antileishmanial drugs with no reported adverse effects in the pancreas of dogs, such as allopurinol, miltefosine, and aminosidine-allopurinol combination, reduction of the meglumine antimoniate dose, or the interruption of treatment should be considered [16, 17, 23, 48].

## Conclusions

The present results demonstrate that *L. infantum* is one of the etiological agents of chronic pancreatitis in dogs, which is associated with a mild to moderate, predominantly granulomatous inflammatory infiltrate. The frequency of detection and load of this parasite are low in the pancreas. The lack of an association of poor body condition and cachexia with pancreatitis and the low frequency of clinical signs commonly associated with pancreatitis suggest that a significant portion of the pancreas is not affected by this parasite. This result and the association of poor body condition and cachexia with a higher frequency of the parasite in the pancreas, as well as *L. infantum* parasitism in the spleen and/or bone marrow of all dogs with pancreatic infection, suggest that poor body condition and cachexia are the results of a more advanced stage of visceral leishmaniasis.

## Data Availability

Data supporting the conclusions of this article are included within the article.

## Abbreviations

ZVL: Zoonotic visceral leishmaniasis
TR DPP®: Rapid dual-path platform assay
ELISA: Enzyme-linked immunosorbent assay
IHC: Immunohistochemistry
EPI: Exocrine pancreatic insufficiency
